# Analysis of the mouse gut microbiome using full-length 16S rRNA amplicon sequencing

**DOI:** 10.1038/srep29681

**Published:** 2016-07-14

**Authors:** Jongoh Shin, Sooin Lee, Min-Jeong Go, Sang Yup Lee, Sun Chang Kim, Chul-Ho Lee, Byung-Kwan Cho

**Affiliations:** 1 Department of Biological Sciences and KI for the BioCentury, Korea Advanced Institute of Science and Technology, Daejeon 34141, Republic of Korea; 2 Laboratory Animal Resource Center, Korea Research Institute of Bioscience and Biotechnology, Daejeon 34141, Republic of Korea; 3 Department of Chemical and Biomolecular Engineering (BK21 Plus program), Korea Advanced Institute of Science and Technology, Daejeon 34141, Republic of Korea; 4 Intelligent Synthetic Biology Center, Daejeon 34141, Republic of Korea

## Abstract

Demands for faster and more accurate methods to analyze microbial communities from natural and clinical samples have been increasing in the medical and healthcare industry. Recent advances in next-generation sequencing technologies have facilitated the elucidation of the microbial community composition with higher accuracy and greater throughput than was previously achievable; however, the short sequencing reads often limit the microbial composition analysis at the species level due to the high similarity of 16S rRNA amplicon sequences. To overcome this limitation, we used the nanopore sequencing platform to sequence full-length 16S rRNA amplicon libraries prepared from the mouse gut microbiota. A comparison of the nanopore and short-read sequencing data showed that there were no significant differences in major taxonomic units (89%) except one phylotype and three taxonomic units. Moreover, both sequencing data were highly similar at all taxonomic resolutions except the species level. At the species level, nanopore sequencing allowed identification of more species than short-read sequencing, facilitating the accurate classification of the bacterial community composition. Therefore, this method of full-length 16S rRNA amplicon sequencing will be useful for rapid, accurate and efficient detection of microbial diversity in various biological and clinical samples.

Microbiotas are complex microbial communities containing hundreds of species-level phylotypes and are found everywhere, from humans (e.g., the microbiota within the gut) to environments. Interestingly, these communities and their genetic blueprint, referred as the microbiome^1^, has been implicated in a variety of human diseases^2^, including inflammatory bowel diseases^3^, type 2 diabetes^4^, and brain abnormalities, such as autism spectrum disorder^5^. Thus, the microbiome has attracted much attention in the medical and healthcare industries, and elucidation of the microbiota composition in the human body is critical for further advancements in our understanding of related diseases and physiological states. In this regard, because species in the same taxonomic units from genus up to phylum play a variety of roles, some may be crucial, others may not be correlated with the phenotype^6^, it is critical to obtain the higher taxonomic resolution to species level for better understanding of the functional effects of microbiota on health and further identifying key players in a specific phenotype^7^.

Until recently, second-generation sequencing has been widely used to assess the composition of the microbial community with higher accuracy and greater throughput than previous methods, enabling the completion of high-profile microbiome projects, such as the Human Microbiome Project^8^. Despite the high-throughput and high sequencing accuracy, second-generation sequencing can produce only a partial (~100–500 bp) sequence of the 16S rRNA gene. Within this technical limitation, researchers have to select the most effective target regions to identify taxa from full-length 16S rRNA gene sequences containing nine hypervariable regions (V1–V9) as phylogenetically informative markers. Additionally, the genetic distance of individual species is related to the similarities among subregions and full-length sequences^9^. Thus, long reads sequencing of the 16s rRNA gene is a promising approach to provide high-resolution analysis of microbial communities at the species level.

Recently, researchers have developed a new nanopore DNA sequencer^10^ (MinION) that has significant advantages, such as long-read output, low cost, portability, and rapid real-time analysis, as compared with other DNA sequencing technologies. Despite the relatively lower accuracy of this method (~80%) compared with other sequencing technologies, nanopore sequencing has been applied to sequencing of eukaryotic^11^, bacterial^12^,^13^,^14^, and viral genomes^15^,^16^. Furthermore, nanopore sequencing has been successfully adapted for cDNA and amplicon sequencing^17^,^18^,^19^,^20^,^21^. However, the plausibility of using this platform to analyze the gut microbiota composition at the species level has not been fully elucidated in comparison with that of short-read sequencing technologies. In this study, we investigated whether the nanopore sequencing is suitable for analyzing the composition of the mouse gut microbiota at the species level and compared the results with those obtained by the short-read sequencing that has so far been widely employed in the field.

## Results

### Short-read sequencing of 16S rRNA V3-V4 region amplicon libraries

**Figure 1:**
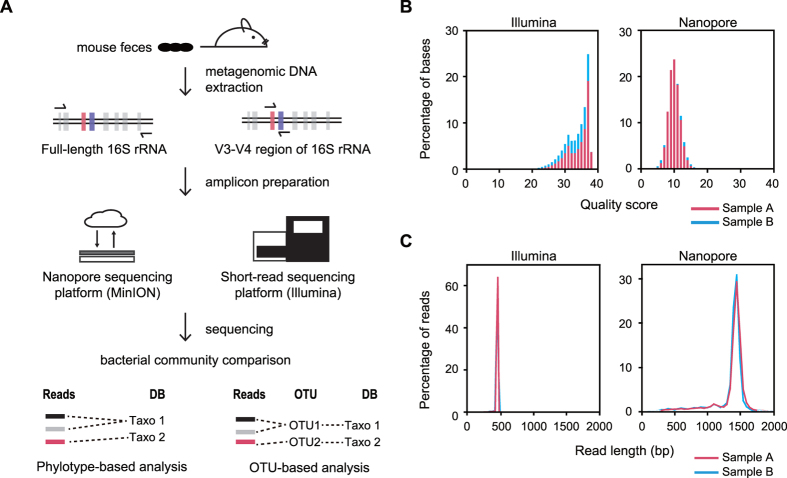
Amplicon sequencing of 16s rDNA gene. (**A**) Schematic workflow to examine the composition of the mouse gut microbiota using the nanopore (MinION) and the short-read (Illumina MiSeq) sequencing. (**B**) The distribution of PHRED quality scores of short-read sequencing data and pass 2D reads of nanopore sequencing data. (**C**) Density plot for length distribution comparison of short-read sequencing data and pass 2D reads of nanopore sequencing data. Each sample is colored separately.

To compare the ability of the short-read and nanopore sequencing platforms to analyze the microbial community, we first prepared short-read sequencing libraries (Illumina platform) from biologically duplicated metagenomic DNAs isolated from the gut microbiome of 50-week-old mice (Fig. 1A). To this end, the V3–V4 hypervariable region (approximately 469 bp) of the 16S rRNA gene, which has been used for taxonomic classification of the microbial community in human microbiome studies, was amplified using a two-step PCR method (see the Materials and Methods for experimental details)^22^. Illumina 250-bp paired-end sequencing of the amplicon targeting the V3–V4 region of the 16S rRNA gene generated 249,593 and 183,029 sequencing reads. Each paired-end read was joined to produce 127,969 and 111,058 reads for samples A and B, respectively, using the QIIME pipeline^23^. Joined reads having less than 75% of their original length were removed in this step. We then performed quality trimming of joined paired sequencing reads (≥Q20), which generated 105,590 and 92,119 sequencing reads. These criteria resulted in a mean read length of about 447 bp, and approximately 83% of sequencing reads were retained for further microbial community analysis (Table 1).

### Nanopore sequencing of full-length 16S rRNA amplicon libraries

At the same time, broad amplification of the full-length 16s rRNA genes from metagenomic DNA samples was achieved using the 16S rRNA gene-specific primers adapted from S-D-bact-0008-c-S20 and S-D-bact-1391-a-A-17 (Fig. 1A)^24^. Using these amplicons, nanopore sequencing libraries were constructed with internal control DNA (see the Materials and Methods for details of nanopore sequencing). The nanopore sequencing generated 101,269 and 33,174 sequencing reads from 923 and 822 pores for samples A and B, respectively (Table 2). The raw data contained the one-dimensional (1D) template, 1D complement, and 2D reads consensus sequence with enhanced accuracy. To obtain high-quality reads, the pass 2D reads were sorted specifically from raw data using the Metrichore 2D base calling program. Integrated information from the template and complement reads could be used for 2D base-calling, which results in a higher mean quality score (Supplemental Fig. S1) and better accuracy than total reads^11^,^25^. Although sample B data (11,078 reads) showed fewer sequencing reads than sample A (36,166 reads), the pass 2D reads were 36% and 33% of total reads with mean quality scores of 9.69 and 9.77, respectively (Fig. 1B and Table 2). The read length had a narrow length distribution, and the mean read length was approximately 1,393 bp, which was nearly the full-length of the 16s rRNA gene (about 1,550 bp; Fig. 1C and Table 2). The unexpected long reads seemed to be the products of concatemers formed at the hairpin adapter ligation step, based on the presence of multiple 16S rRNA gene-specific primer binding sites in their sequences.

The accuracy of the nanopore sequencing was computed based on the coverage (≥80%) of the sequencing reads of the internal control DNA (DNA CS) against the reference sequence using the LAST aligner (version 658)^26^. The LAST alignment algorithm, using adaptive seeds for alignment, has been used to align nanopore sequencing reads to references^27^. Sequencing accuracy was defined as the number of matching nucleotides divided by the total number of matches, mismatches, insertions, and deletions^26^. Consequently, nanopore sequencing exhibited an average accuracy of 79.6%, with 9.0% mismatches, 6.4% insertions, and 5.0% deletions (Supplemental Fig. S2). This accuracy was similar to that reported in previous studies (70–80%)^26^,^28^.

### Sequencing data analysis

**Figure 2:**
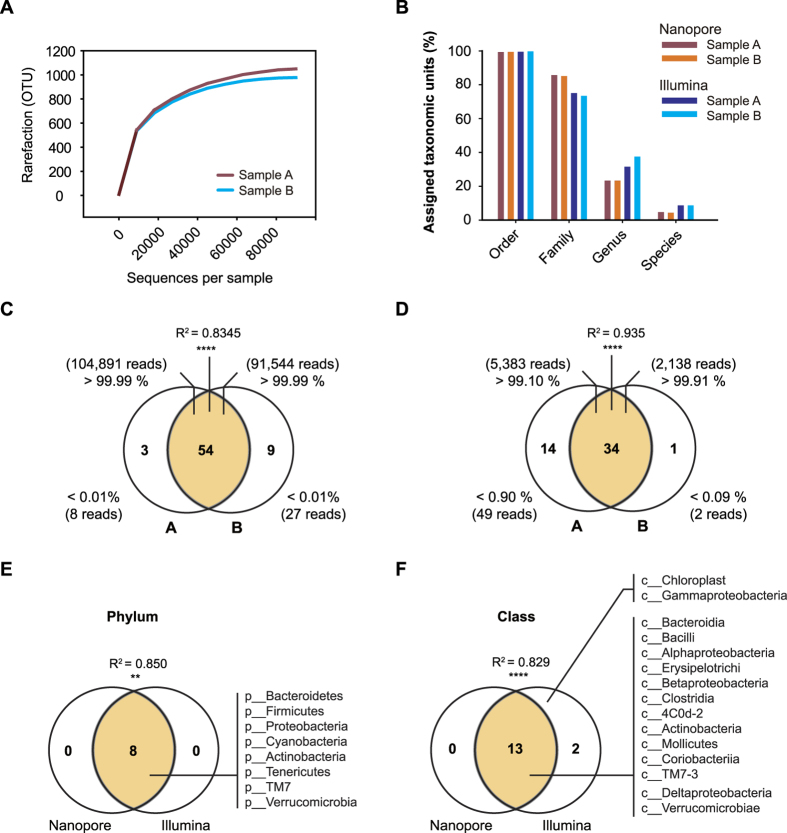
Statistical comparison between short-read and nanopore sequencing data. (**A**) Rarefaction curves of mouse fecal samples based on short-read sequencing (Illumina). Total OTUs were generated by 3% distances. Total sample richness estimates were calculated by the observed OTUs. (**B**) Percentage of taxonomic units assigned as reads at the order, family, genus, and species levels. (**C**) Venn diagram showing the shared and specific phylotypes between Illumina A and B data. The Spearman rank correlation test (R^2^ = 0.8345, *p* < 0.0001) showed the significance of relationships between duplicates. Asterisks indicate the significance of the pairing (****p < 0.0001) (**D**) Two-way Venn diagram depicting the number of shared and specific phylotypes between nanopore A and B data. Percentages show the proportion of aligned reads corresponding to each phylotype per total reads. The results of Spearman rank correlation test (R^2^ = 0.9350, *p* < 0.0001) showed the significance of relationships between duplicates. Asterisks indicate the significance of the pairing (****p < 0.0001). (**E,F**) Venn diagram showing the shared and specific taxonomic units at the (**E**) phylum and (**F**) class levels between nanopore and Illumina sequencing data. The Spearman rank correlation test showed the significance of the relationship. Asterisks indicate the significance of the pairing (** 0.01 < *p* < 0.001, ****p < 0.0001).

The short-read sequencing data sets were then analyzed using the operational taxonomic unit (OTU) approach. To this end, the QIIME pipeline was used to cluster the 16S rRNA gene sequences based on their similarity; this approach has been widely used for microbial community analysis^8^,^23^. Within these data, 104,899 and 91,571 sequencing reads were clustered into 1,055 and 978 OTUs from the data for samples A and B, respectively, at the 0.03 dissimilarity threshold (Table 3). OTU taxonomy was then determined using the Ribosomal Database Project classifier retrained toward the Greengenes database of 13_8 version^29^. The richness of gut microbiota was estimated by the rarefaction curve, which showed similar patterns between biological duplicates (Fig. 2A and Supplemental Fig. S3).

On the other hand, the microbiota composition was determined based on the nanopore sequencing data obtained with the phylotyping approach (taxonomy-supervised analysis), which allocates sequences directly into taxonomic bins based on their similarity. Since computational clustering based on sequence similarity is not required for this approach^30^, it is more tolerant to unnatural variants often observed in OTU-based approach^31^. To assign the taxonomic units, 5,432 and 2,140 of the pass 2D reads were aligned to the GreenGene reference (13_8 version) with a mean length of 1,312 bp and 1,308 bp using LAST, respectively (Table 3). With this method, 15% (5,432 of 36,166) and 19% (2,140 of 11,174) of the pass 2D reads were aligned to the taxonomic reference, and a large portion of nonaligning reads remained unidentified. However, assignments of taxonomic units were significantly similar between duplicates (Fig. 2B).

From the short-read sequencing data, we observed 54 phylotypes shared by two biological duplicates (>99.99% of sequencing reads of each sample). Only three and nine phylotypes were detected from samples A and B, respectively, with less than 0.01% of sequencing reads (Fig. 2C and Supplemental Table S1). Similarly, 34 phylotypes were identified from both nanopore sequencing data sets (Spearman’s rank correlation, R^2^ = 0.935, *p* < 0.0001) with 99.1% and 99.9% of the pass 2D reads, respectively (Fig. 2D and Supplemental Table S2). Fourteen and one phylotypes were assigned by 0.9% (49 of 5,432) and 0.09% (2 of 2,140) of the pass 2D reads, respectively. Although sample-specific phylotypes were observed at a negligible level, and a relatively high error rate (20.4%) was determined from the nanopore sequencing reads, high reproducibility and correlation were achieved between the biological duplicates. Thus, 34 major phylotypes were reproducibly detected using nanopore amplicon sequencing.

### Comparison of microbial composition determined by two sequencing platforms

Next, we compared the microbial compositions determined using the two sequencing platforms. Both platforms identified eight bacteria phyla and 13 bacteria classes (Fig. 2E,F). Statistically significant similarity was observed in the relative proportions of members of the major phyla (Spearman’s rank correlation, R^2^ = 0.850, *p* = 0.003) and classes (Spearman’s rank correlation, R^2^ = 0.829, *p* < 0.0001) between short-read and nanopore sequencing data.

**Figure 3:**
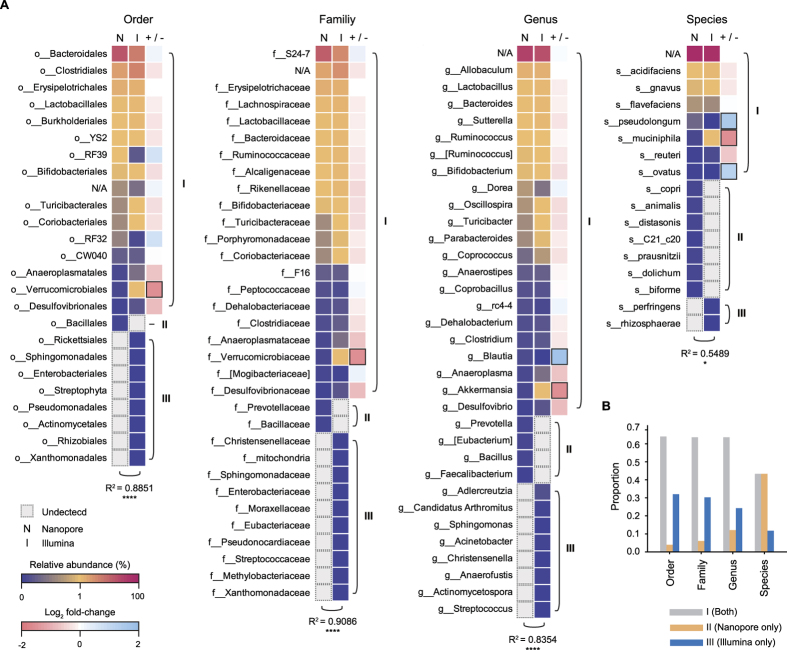
Comparison of mouse gut microbiota compositions between two sequencing platforms at deeper classifications (order to species). (**A**) Heat map for the mean relative abundances for the two platforms at the order, family, genus, and species levels. Nanopore and Illumina columns are colored with relative abundances ranging from 0% to 100% according to the color key in the lower left corner of the figure. The +/− column is colored with log_2_ fold-changes ranging from −2 to 2 according to the color key in the lower left corner of the figure. The black border indicates significantly different abundance deviation (log_2_ fold-change, lower than −1 or higher than 1). The taxonomic units were categorized as I, II, and III according to whether they were detected by Illumina only (I), nanopore only (II), or both platforms (III). The Spearman rank correlation test (R^2^) showed the significance of relationships between the two platforms. Asterisks indicate the significance of the pairing (*p < 0.05, ****p < 0.0001). (**B**) Proportion of groups (I–III) at the levels of taxonomic classification from order to species.

The relative abundances of microbial compositions detected from the two sequencing platforms were then depicted using heat maps at the order, family, genus, and species levels (Fig. 3A). All taxonomic units were classified into three groups based on whether they were detected by the short-read sequencing platform only (group III), the nanopore sequencing platform only (group II), or both platforms (group I). The relative abundances of the most dominant phylotype in nanopore and short-read sequencing data were quite similar. In the short-read sequencing data, taxonomic units (39 units) with high abundance (>0.5%) were observed only in group I. On the other hand, taxonomic units (66 units) with low abundance (<0.5%) were observed in all groups. Although one phylotype (o__*Verrucomicrobiales*; f__*Verrucomicrobiaceae*; g__*Akkermansia*; s__*muciniphila*) and three taxonomic units (g__*Blautia*, s__*pseudolongum*, and s__*ovatus*) showed different abundance deviations (log_2_ fold-change: −1.58–1.81), the others (89%) had no significant differences (log_2_ fold-change: >−1 or <1) in group I (Fig. 3A). All genera-based taxonomic units detected from the nanopore sequencing data were similar to the previous mouse gut microbiota analysis^32^,^33^,^34^,^35^. For example, *Prevotella* was reported to be present at relatively low abundance in the mouse gut microbiota^32^. Furthermore, all bacterial species detected from groups I and II have also been identified in the mouse gut microbiota^36^,^37^,^38^,^39^,^40^,^41^,^42^,^43^. For example, the compositions of *Lactobacillus reuteri* and *Bifidobacterium animalis* are associated with animal obesity^44^, and *Bacteroides ovatus* has been reported to provide XyG catabolism to the host as a common gut symbiont^38^. Overall, the bacterial compositions were significantly similar between the two platforms at the order (Spearman’s rank correlation, R^2^ = 0.8851, *p* < 0.0001), family (Spearman’s rank correlation, R^2^ = 0.9086, *p* < 0.0001), genus (Spearman’s rank correlation, R^2^ = 0.8354, *p* < 0.0001), and species levels (Spearman’s rank correlation, R^2^ = 0.5389, *p* = 0.0124). Thus, comparative analysis of the microbial composition independently profiled by nanopore and short-read sequencing platforms showed that nanopore sequencing was capable of determining the correct microbial composition up to the species level.

### Species detection using full-length 16S rDNA amplicon sequencing

**Figure 4:**
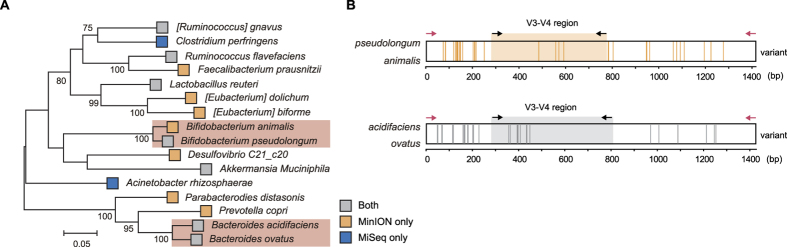
Phylogenetical analysis of mouse gut microbiota. (**A**) Maximum-likelihood phylogenetic tree of 16 species identified in this study. The tree was generated in MEGA6^55^. Reference sequences were obtained from the GreenGene (13_8) database. The clustering of the sequences was tested by a bootstrap approach with 1,000 repeats, and bootstrap values below 70 were clipped. The red box indicates the species separated from their genus. (**B**) Detected variants between two 16S rRNA gene sequences of the separated species are represented as a vertical line on the 16S rDNA sequences. Variants were defined as nucleotide present in less than 50% of aligned position frequencies. The black and red arrows indicate the binding positions of primer sets for the amplification of V3–V4 regions and nearly full-length regions on 16S rDNA sequences, respectively.

We also observed the different proportions of groups at different taxonomic resolutions (Fig. 3B). According to the taxonomic resolution from order to species, the relative proportions of groups I (both sequencing platforms) and III (short-read sequencing only) were decreased. This observation may reflect in the fact that the insufficient read length or sequencing depth was used to analyze the microbial composition. In contrast, the relative proportion of group II (nanopore sequencing only) was increased. In this regard, we hypothesized that long reads generated by nanopore sequencing spanned multiple variable regions and could better separate the microbial composition than Illumina sequencing due to the nearly full-length 16s rDNA reads. To identify the effects of long reads on the microbial composition analysis at the species level, we performed phylogenetic analysis of combined datasets by priority and identified 16 phylogenetically distinct species distributed in 13 genera (Fig. 4A). As a result, four species (*Bacteroides acidifacies*, *Bacteroides ovatus*, *Bifidobacterium animalis*, and *Bifidobacterium psudolongum*) were separated from their genera *Bacteroides* and *Bifidobacterium* in the phylogenetic tree. Interestingly, the taxonomic separation of *Bifidobacterium* genus was only observed in the nanopore sequencing data, unlike the *Bacteroides* genus. This indicated that *Bacteroides acidifacies* and *Bacteroides ovatus* could be separated by OTUs defined as a cluster of short reads with 97% similarity, whereas *Bifidobacterium animalis* could not (Fig. 4A).

For further investigation, we compared the divergence of each reference 16S rRNA sequence. Each taxonomic reference 16S rRNA sequence corresponding to the species was extracted from the aligned data of nanopore reads, followed by aligning them using the ClustalW multiple alignment algorithm^45^. All variants of each reference 16S rRNA sequence were determined by detecting the allele frequencies (less than 50%) from the multiple aligned taxonomic references. Nine variants of V3–V4 regions (total 37 variants) were observed between *Bacteroides acidifacies* and *Bacteroides ovatus*, whereas four variants (total 39 variants) were detected between *Bifidobacterium* species (Fig. 4B). The variants of the 16S rRNA gene between *Bifidobacterium animalis* and *Bifidobacterium pseudolongum* were enriched, particularly in the V1–V2 regions, compared with other variable regions. As expected, however, other phylogenetically similar species within different genera had more variants than species within the same genus (Supplemental Fig. S4). Taken together, these findings suggested that the V3–V4 region was insufficient for analysis of the microbial community composition at the species level and that full-length 16S rRNA sequencing had the advantage of covering multiple variable regions of 16s rRNA genes.

## Discussion

To investigate the relationship between host and microbiota, several methods have been employed for the detection of microbial community composition from natural and clinical samples. In particular, the composition of the gut microbiota has been elucidated using both metagenomic and 16S rRNA amplicon sequencing approaches, the latter of which is most commonly used^8^. Currently, metagenomic and metatranscriptomic sequencing of microbiota are actively applied with increased sequencing depth to improve our understanding of the microbial community with better resolution^46^,^47^. In addition, despite the limited sequencing output level, long reads produced by PacBio sequencing platform have begun to demonstrate their potential to provide accurate analysis of the microbial community composition^48^.

Here, we examined the potential of a third-generation sequencer, MinION, for identification of the microbiome composition in mouse fecal samples. Although long reads generated from the nanopore sequencer were found to have relatively higher error rates compared with other platforms, sequencing reads of nearly full-length 16S rRNA could provide more accurate taxonomy assignment of the entire microbial community than short sequencing reads obtained from the hypervariable region (V3-V4) amplicon library. Aside from this result, high proportion (81–85%) of unmapped pass 2D reads was observed due to the current error rate of MinION platform and may directly give rise to assigning an unrelated organism or low-throughput. Consequently, the sequencing accuracy may influence the further analysis of the microbiome composition.

When comparing nanopore sequencing results from the two biological duplicates, however, most abundant taxonomic units (89%) did not exhibit significant differences (log_2_ fold-change, lower than −1 or higher than 1), except one phylotype and three taxonomic units, and the sequencing data were highly similar (R^2^ = 0.829–0.909) at all taxonomic resolutions, except the species level. At the species level, we observed that nanopore sequencing data had better resolution than the short-read sequencing data. In particular, we obtained *Bifidobacterium animalis* and *Bifidobacterium pseudolongum*, which have been reported as key members of the gut microbial community^49^, at the species level. This result showed that the identification of some strains at the species level could be impossible when using a partial region of the 16S rRNA (e.g., *Neisseria meningitidis* and *Neisseria lactamica*). Therefore, full-length 16s rRNA sequencing provided higher taxonomic resolution than second-generation sequencing. Moreover, within the clinical setting, the long reads from the 16S rRNA sequence may be promising. According to our data, if appropriate clinical standard references are constructed, such references could be used as specific microbial markers to profile individual microbiomes without the computational burden of forming OTUs.

With long reads, nanopore sequencing (e.g., MinION) has been shown to have significant advantages, such as small size, low cost, rapid library construction (<3 h), and real-time detection. These advantages suggest the potential for identification of members of a microbiota community *in situ*. Additionally, nanopore sequencing can eliminate sample storage steps; such steps may cause loss of important species used as biomarkers (e.g., *Bacteroidetes*) due to long-term freezing of samples^50^. Similarly, previous studies have supported that the MinION sequencer can be used as a real-time molecular diagnostic device^14^,^16^. Current limitations, such as accuracy and throughput, will be resolved by advanced nanopore chemistry. For example, the SQK-MAP-006 sequencing kit (Oxford Nanopore Technologies), which was used in this paper, provides improved results with 2-fold faster sequencing speed, new hairpin-motor adaptors for high 2D yield, and advanced modeling of base-calling algorithm (6-mers) compared with previous chemistries (SQK–MAP005 and SQK–MAP005.1). Also, next generation chemistry (R9, Oxford Nanopore Technologies) contains a new pore protein, such as the CsgG protein^51^,^52^, which is predicted to provide more optimal sensing regions for a DNA strand as compared with MspA and α-hemolysin. It will be helpful in improving the sequencing accuracy and throughputs of the nanopore sequencing platform. Further, automatic devices (e.g. VolTRAX™, Oxford Nanopore Technologies) and improved library construction methods (e.g. adaptor-charged transposase mediated library preparation) will be used as the multiplex, fast, and high-throughput methods for preparing the nanopore sequencing library in the near future.

In conclusion, the full-length 16S rRNA amplicon sequencing with a nanopore sequencer allows rapid, accurate and efficient determination of the microbial diversity at the species level as successfully demonstrated for determining microbiome composition. We anticipate that this method will broaden the utility of microbiome composition analysis for biological, clinical, and environmental origins.

## Methods

### Metagenomic DNA extraction

Microbial metagenomic DNA was extracted with a PowerSoil DNA Isolation Kit (MoBio, Carlsbad, CA, USA). Snap-frozen fecal samples stored at −80 °C were added to PowerBead tubes and treated as described in the manufacturer’s instructions. The tubes containing the pretreated samples were placed into a benchtop homogenizer FastPrep-24 5G (MP Biomedicals, Santa Ana, CA, USA) and disrupted for 40 s, three times, with a 1-min rest period. The machine speed setting was 6 m/s, and a QuickPrep adapter was used. The concentration of the extracted DNA was measured with a Nanodrop 2000 (Thermo Scientific, Waltham, MA, USA).

### Nanopore sequencing library construction

16S-specific primers adapted from S-D-bact-0008-c-S20 and S-D-bact-1391-a-A-17 were used for broad-taxonomic range amplification of the bacterial 16S rRNA gene^24^. For polymerase chain reaction (PCR) amplification using Phusion High-Fidelity polymerase (Thermo Scientific), 3 μg of the 16S-specific primers were added to the 30 ng of metagenomic DNA. The amplification was monitored with SYBR Green gel staining solution (Invitrogen, Grand Island, NY, USA) on a CFX96 Real-Time PCR Detection System (Bio-Rad, Hercules, CA, USA) and stopped at the beginning of the saturation point to reduce PCR bias. The PCR conditions were as follows: 98 °C for 30 s; 15 cycles of 98 °C for 10 s, 47 °C for 30 s, and 72 °C for 60 s; followed by 72 °C for 5 min. PCR products were purified using a MinElute Gel Extraction kit (Qiagen, Venlo, Netherlands). The amount of recovered DNA was quantified using a Qubit 3.0 fluorometer (Life Technologies, Carlsbad, CA, USA), and 300 ng of purified amplicon DNA with 5 μL of internal control DNA (DNA CS from the SQK-MAP006 kit) was used as input for generation of MinION-compatible libraries. The amplicons were end repaired using the NEBNext End Repair module (NEB, Ipswich, MA, USA). Subsequently, the end-repaired amplicon was dA-tailed using the NEBNextdA-tailing module (NEB) at 37 °C for 10 min. Then, 30 μL dA-tailed DNA, 50 μL Blunt/TA ligase master mix (NEB), 10 μL of Adapter Mix (Oxford Nanopore Technologies, Oxford, UK), and 2 μL HP adapter (Oxford Nanopore Technologies) were added and incubated at room temperature for 10 min. The adaptor-ligated libraries were purified using MyOne C1-beads (Thermo Scientific), eluted in 25 μL elution buffer (Oxford Nanopore Technologies), and incubating at 37 °C for 10 min.

### Nanopore sequencing and base-calling

Each nanopore sequencing library was run on a FLO-MAP103 flow cell after performing platform QC analysis. The FLO-MAP103 flow cell was primed twice with a mixture of Fuel Mix (Oxford Nanopore Technologies). Ten microliters of the amplicon library (8 ng) was diluted in 75 μL of 2× running buffer with 61 μL nuclease-free water and 4 μL Fuel Mix. A 48 h sequencing protocol was initiated using the MinION control software, MinKNOW (version 0.50.2.15). Raw FAST/HDF files were base-called by the Metrichor agent two-dimensional (2D) base-calling workflow. Pass 2D reads were converted for downstream analysis into a FASTA format using *poretools*^53^.

### Illumina sequencing

A 16S rRNA sequencing library was constructed according to the 16S metagenomics sequencing library preparation protocol (Illumina, San Diego, CA, USA) targeting the V3 and V4 hypervariable regions of the 16S rRNA gene, as were also used by the Human Microbiome Project^8^. KAPA HiFi HotStart ReadyMix (KAPA Biosystems, Wilmington, MA, USA) and Agencourt AMPure XP system (Beckman Coulter Genomics, Brea, CA, USA) were used for PCR and purification of the PCR product, respectively. The initial PCR was performed with 12 ng template DNA using region-specific primers shown to have compatibility with Illumina index and sequencing adapters (forward primer: 5′-TCGTCGGCAGCGTCAGATGTGTATAAGAGACAGTCGTCGGCAGCGTCAGATGTGTATAAGAGACAGCCTACGGGNGGCWGCAG-3′; reverse primer: 5′-GTCTCGTGGGCTCGGAGATGTGTATAAGAGACAGGTCTCGTGGGCTCGGAGATGTGTATAAGAGACAGGACTACHVGGGTATCTAATCC-3′). After magnetic bead-based purification of PCR products, the second PCR was performed using primers from a Nextera XT Index Kit (Illumina) with a limited cycle. Subsequently, purified PCR products were visualized using gel electrophoresis and quantified with a Qubit dsDNA HS Assay Kit (Thermo Scientific) on a Qubit 3.0 fluorometer. The pooled samples were run on an Agilent 2200 TapeStation (Agilent Technologies, Santa Clara, CA, USA) for quality analysis prior to sequencing. The sample pool (4 nM) was denatured with 0.2 N NaOH, diluted further to 4 pM, and combined with 20% (v/v) denatured 4 pM PhiX, prepared following Illumina guidelines. Samples were sequenced on the MiSeq sequencing platform (Illumina) using a 2 × 250 cycle V3 kit, following standard Illumina sequencing protocols.

### Nanopore sequencing data analysis

For sequencing analysis, 2D reads were aligned against the GreenGenes 13_8 reference sequences using the LAST aligner v.658 with the following parameters: -q 1 -a 1 -b 1(match score of 1, gap opening penalty of 1, and gap extension penalty of 1). Alignment information was converted to *maf* files with *maf-convert*, which was packaged in LAST, to build *.axt* files. Samtools version 0.1.19^54^ was used to produce *.bam* files and alignment information from *.axt* files. For each read, the highest scoring alignment was retained and assigned with the taxonomic id of corresponding GreenGene reference sequences. The low abundance data of single mapped reads were discarded when considering assigned taxonomic units to reduce spurious taxonomic units. To assess the nanopore sequencing accuracy, total 2D reads were aligned against the phage lambda sequences (NC_001416.1), which were used as the reference for DNA CS with LAST (version 658) using the following parameters: -q 1 -a 1 -b 1. Samtools (version 0.1.19) and count-errors.py, obtained from the *poretools* repository^53^, were used to call variants in the region spanned by at least 80% of the strands. The mismatch positions in the sequencing reads aligned to the reference sequence were counted to measure the insertions, deletions, and substitutions. The sequencing accuracy was calculated as the number of matching nucleotides divided by the sum of matches, mismatches, insertions, and deletions aligned against the reference sequence.

### Illumina sequencing data analysis

The QIIME pipeline (version 1.9.1) was used to process and filter multiplexed sequence reads. OTUs were clustered against GreenGenes 13_8 reference sequences, and reads failing to hit the reference were subsequently clustered *de novo* at the 97% similarity level using the UCLUST greedy algorithm. Chimeric sequences were identified by the UCHIME algorithm included in the free version of USEARCH61 and removed. OTU sequences were aligned using PYNAST. OTU taxonomy was determined using the Ribosomal Database Project classifier retrained toward the GreenGenes database. To avoid biases generated by differences in sequencing depth and removal of plastid sequences, the OTU table was rarified to an even depth of 90,000 sequences per sample in comparisons of all sample types.

### Animal Experiments

Fifty-week-old male C57BL/6J mice were housed in a room maintained at a condition with 12 h light/dark cycle at 22 ± 2 °C, and allowed free access to unlimited food and water in a specific pathogen-free facility of the Korea Research Institute of Bioscience and Biotechnology (KRIBB, Daejeon, Korea). All mice were humanely euthanized by CO_2_ asphyxiation and then, the feces samples were directly obtained from the each mouse colon following necropsy. All animal experiments were approved by the Institutional Animal Use and Care Committee of KRIBB and were performed in accordance with the Guide for the Care and Use of Laboratory Animals published by the US National Institutes of Health (NIH Publication, 8th Edition, 2011).

## Additional Information

**How to cite this article**: Shin, J. *et al*. Analysis of the mouse gut microbiome using full-length 16S rRNA amplicon sequencing. *Sci. Rep.* **6**, 29681; doi: 10.1038/srep29681 (2016).

## Supplementary Material

Supplementary Information

## Figures and Tables

**Table 1:** Statistics of short-read sequencing (Illumina) data.

Sample	Read count	Joined reads	Quality filtered reads (≥Q20)	Reads length (bp)			Total number of bases (bp)
				Min	Mean	Max	
A	249,593	127,969	105,590	332	446.4	502	47,130,530
B	183,029	111,058	92,119	336	447.5	496	41,219,834

**Table 2:** Statistics of nanopore sequencing (MinION) data.

Sample	Active pore#	Total reads	Pass 2D reads (%)	Reads length (bp)			Quality score (PHRED)		
				Min	Mean	Max	Min	Mean	Max
A	923	101,269	36,166 (36%)	172	1,391	55,287	9.00	9.69	12.27
B	822	33,174	11,078 (33%)	111	1,393	73,809	9.00	9.77	13.02

**Table 3:** Microbial community analysis using short-read (Illumina) and nanopore (MinION) sequencing.

Sample	Read count	OTU count	Reads assigning taxonomic labels (coverage %)			
			Order	Family	Genus	Species
A (Illumina)	104,899	1,055	104,389 (99.5%)	78,738 (75.1%)	33,061 (31.5%)	9,082 (8.7%)
B (Illumina)	91,571	978	91,302 (99.7%)	67,229 (73.4%)	34,363 (37.5%)	7,936 (8.7%)
A (MinION)	5,432	N/D	5,396 (99.3%)	4,656 (85.7%)	1,268 (23.3%)	258 (4.7%)
B (MinION)	2,140	N/D	2,145 (99.4%)	1,822 (85.1%)	498 (23.3%)	91 (4.3%)
