# Supplementary material for: Analysis of the mouse gut microbiome using full-length 16S rRNA amplicon sequencing
Source: Sci Rep. 2016 Jul 14;6:29681. doi: 10.1038/srep29681 (PMC4944186; doi:10.1038/srep29681)
Supplement: Supplementary Information [file srep29681-s1.pdf]

Analysis of the mouse gut microbiome using full-length 16S rRNA amplicon sequencing

Jongoh Shin[1,¶], Sooin Lee[1,¶], Min-Jeong Go[2], Sang Yup Lee[3], Sun Chang Kim[1,4], Chul-Ho Lee[2], and Byung-Kwan Cho[1,4,*]

[1]Department of Biological Sciences and KI for the BioCentury, Korea Advanced Institute of Science and Technology, Daejeon 34141, Republic of Korea

[2]Laboratory Animal Resource Center, Korea Research Institute of Bioscience and Biotechnology, Daejeon 34141, Republic of Korea

[3]Department of Chemical and Biomolecular Engineering, Korea Advanced Institute of Science and Technology, Daejeon 34141, Republic of Korea

[4]Intelligent Synthetic Biology Center, Daejeon 34141, Republic of Korea

Supplementary Data

Figures S1-S4

Tables S1-S2

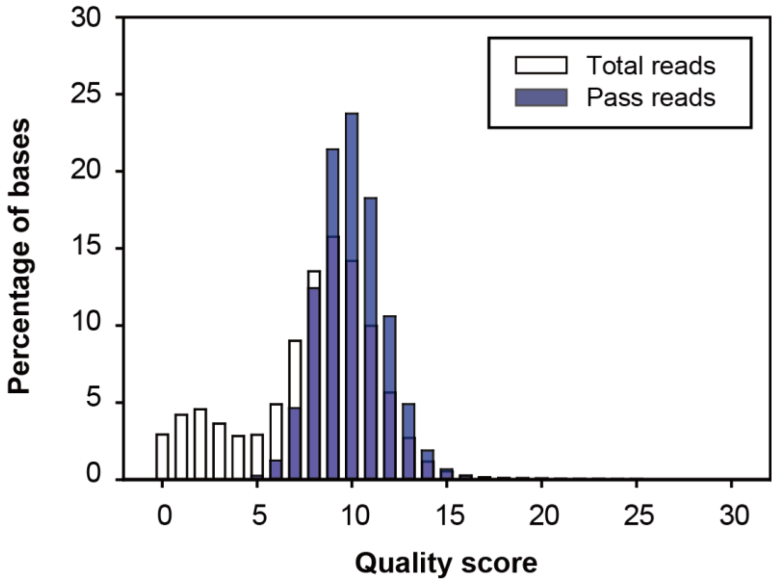

Supplemental Figure S1. MinION™ quality score distribution. The distribution of PHRED quality score of MinION™ raw data is plotted on X-axes and percentage of bases on Y-axes for “Total reads” and “Pass 2D reads”.

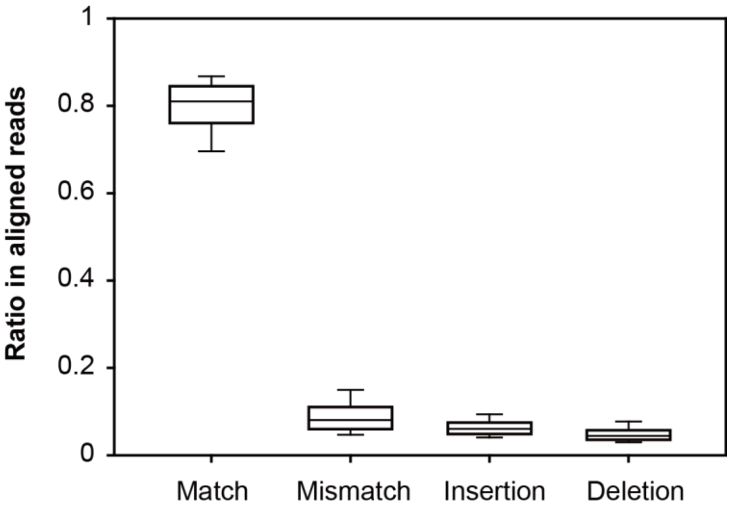

Supplemental Figure S2. Comparison of ratios of match, mismatch, insertion and deletion. The proportion of accuracy was 0.796 in aligned reads. Mismatch, insertion and deletion ratio in aligned reads was 0.090, 0.064 and 0.050, respectively. Box plot show interquartile range with 25th and 75th percentiles; median is the solid line in each box.

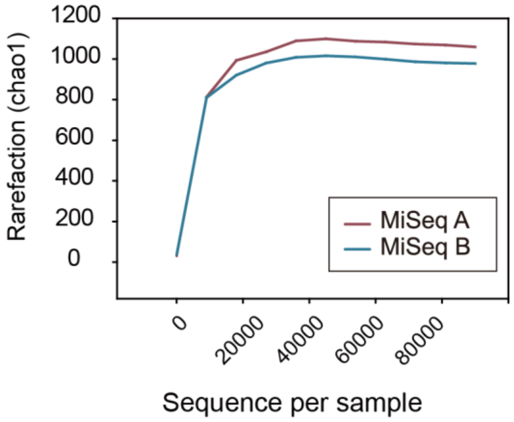

Supplemental Figure S3. Rarefaction curves of mouse fecal samples based on Illumina MiSeq sequencing. A total OTUs were generated by 3% distances. Total sample richness estimates were calculated by richness estimators Chao1.

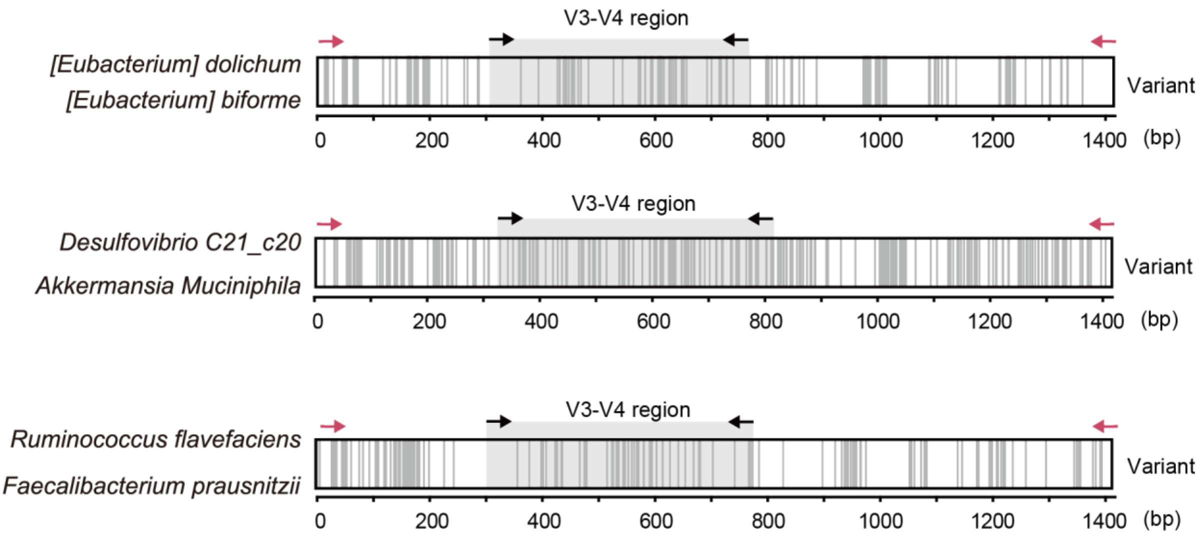

Supplemental Figure S4. Detected variants between 16S rRNA gene sequences from two phylogenetically related species were represented with vertical line on 16s rDNA sequences. Variants were defined as nucleotide presents in less than 50% of aligned position frequency. The black and red arrow indicates binding position of primer sets for the amplification of V3–V4 regions and nearly full-regions on 16s rDNA sequences, respectively.

Supplemental Table S1. Relative abundance of phylotypes detected by MiSeq sequencer.

Taxonomy	MiSeq A (%)	MiSeq B (%)
k__Bacteria;p__Bacteroidetes;c__Bacteroidia;o__Bacteroidales;f__S24-7;	32.808	25.582
k__Bacteria;p__Firmicutes;c__Clostridia;o__Clostridiales;	20.494	25.279
k__Bacteria;p__Firmicutes;c__Erysipelotrichi;o__Erysipelotrichales;f__Erysipelotrichaceae;g__Allobaculum;	5.421	10.833
k__Bacteria;p__Bacteroidetes;c__Bacteroidia;o__Bacteroidales;f__Bacteroidaceae;g__Bacteroides;s__acidifaciens;	5.105	2.504
k__Bacteria;p__Bacteroidetes;c__Bacteroidia;o__Bacteroidales;f__Rikenellaceae;	4.244	4.329
k__Bacteria;p__Proteobacteria;c__Betaproteobacteria;o__Burkholderiales;f__Alcaligenaceae;g__Sutterella;	3.627	4.076
k__Bacteria;p__Cyanobacteria;c__4C0d-2;o__YS2;	3.531	0.774
k__Bacteria;p__Firmicutes;c__Bacilli;o__Lactobacillales;f__Lactobacillaceae;g__Lactobacillus;	3.195	5.372
k__Bacteria;p__Actinobacteria;c__Actinobacteria;o__Bifidobacteriales;f__Bifidobacteriaceae;g__Bifidobacterium;	2.257	2.109
k__Bacteria;p__Firmicutes;c__Clostridia;o__Clostridiales;f__Lachnospiraceae;g__[Ruminococcus];s__gnavus;	2.064	1.149
k__Bacteria;p__Firmicutes;c__Clostridia;o__Clostridiales;f__Lachnospiraceae;	1.962	1.819
k__Bacteria;p__Firmicutes;c__Clostridia;o__Clostridiales;f__Ruminococcaceae;g__Oscillospira;	1.864	1.554
k__Bacteria;p__Firmicutes;c__Bacilli;o__Turicibacterales;f__Turicibacteraceae;g__Turicibacter;	1.510	1.282
k__Bacteria;p__Bacteroidetes;c__Bacteroidia;o__Bacteroidales;f__Porphyromonadaceae;g__Parabacteroides;	1.429	0.510
k__Bacteria;p__Firmicutes;c__Clostridia;o__Clostridiales;f__Ruminococcaceae;	1.383	1.257

Taxonomy	MiSeq A (%)	MiSeq B (%)
k__Bacteria;p__Actinobacteria;c__Coriobacteriia;o__Coriobacteriales;f__Coriobacteriaceae;	1.125	1.272
k__Bacteria;p__Firmicutes;c__Clostridia;o__Clostridiales;f__Ruminococcaceae;g__Ruminococcus;s__flavefaciens;	1.077	0.149
k__Bacteria;p__Firmicutes;c__Clostridia;o__Clostridiales;f__Lachnospiraceae;	0.901	1.195
k__Bacteria;p__Firmicutes;c__Clostridia;o__Clostridiales;f__Lachnospiraceae;g__Coprococcus;	0.738	0.714
k__Bacteria;p__Firmicutes;c__Erysipelotrichi;o__Erysipelotrichales;f__Erysipelotrichaceae;	0.717	0.123
k__Bacteria;p__Tenericutes;c__Mollicutes;o__Anaeroplasmatales;f__Anaeroplasmataceae;g__Anaeroplasma;	0.673	0.103
k__Bacteria;p__Firmicutes;c__Clostridia;o__Clostridiales;f__Ruminococcaceae;g__Ruminococcus;	0.463	0.745
Unassigned;	0.416	0.268
k__Bacteria;p__Firmicutes;c__Clostridia;o__Clostridiales;f__Lachnospiraceae;g__Anaerostipes;	0.413	0.106
k__Bacteria;p__Firmicutes;c__Clostridia;o__Clostridiales;f__Lachnospiraceae;g__Dorea;	0.337	0.512
k__Bacteria;p__Firmicutes;c__Erysipelotrichi;o__Erysipelotrichales;f__Erysipelotrichaceae;g__Coprobacillus;	0.303	0.005
k__Bacteria;p__TM7;c__TM7-3;o__CW040;f__F16;	0.276	0.220
k__Bacteria;p__Verrucomicrobia;c__Verrucomicrobiae;o__Verrucomicrobiales;f__Verrucomicrobiaceae;g__Akkermansia;s__muciniphila;	0.273	4.733
k__Bacteria;p__Tenericutes;c__Mollicutes;o__RF39;	0.252	0.162
k__Bacteria;p__Firmicutes;c__Clostridia;o__Clostridiales;f__Dehalobacteriaceae;g__Dehalobacterium;	0.238	0.109
k__Bacteria;p__Firmicutes;c__Clostridia;o__Clostridiales;f__Peptococcaceae;g_	0.154	0.002

Taxonomy	MiSeq A (%)	MiSeq B (%)
_rc4-4;		
k__Bacteria;p__Proteobacteria;c__Alphaproteobacteria;o__RF32;	0.140	0.037
k__Bacteria;p__Firmicutes;c__Clostridia;o__Clostridiales;f__Clostridiaceae;g__Clostridium;	0.104	0.250
k__Bacteria;p__Firmicutes;c__Bacilli;o__Lactobacillales;f__Lactobacillaceae;g__Lactobacillus;s__reuteri;	0.086	0.085
k__Bacteria;p__Proteobacteria;c__Alphaproteobacteria;	0.061	0.020
k__Bacteria;p__Actinobacteria;c__Coriobacteriia;o__Coriobacteriales;f__Coriobacteriaceae;g__Adlercreutzia;	0.044	0.210
k__Bacteria;p__Proteobacteria;c__Deltaproteobacteria;o__Desulfovibrionales;f__Desulfovibrionaceae;g__Desulfovibrio;	0.043	0.322
k__Bacteria;p__Firmicutes;c__Clostridia;o__Clostridiales;f__Peptococcaceae;	0.035	0.004
k__Bacteria;p__Firmicutes;c__Clostridia;o__Clostridiales;	0.034	0.031
k__Bacteria;p__Firmicutes;c__Clostridia;o__Clostridiales;f__Clostridiaceae;	0.033	0.015
k__Bacteria;p__Firmicutes;c__Clostridia;o__Clostridiales;f__Christensenellaceae;	0.031	0.007
k__Bacteria;p__Firmicutes;c__Clostridia;o__Clostridiales;f__[Mogibacteriaceae];	0.025	0.025
k__Bacteria;p__Firmicutes;c__Clostridia;o__Clostridiales;f__Clostridiaceae;g__Clostridium;s__perfringens;	0.024	0.004
k__Bacteria;p__Firmicutes;c__Clostridia;o__Clostridiales;f__Clostridiaceae;g__Candidatus Arthromitus;	0.022	0.029
k__Bacteria;p__Bacteroidetes;c__Bacteroidia;o__Bacteroidales;f__Bacteroidaceae;g__Bacteroides;	0.018	0.013
k__Bacteria;p__Actinobacteria;c__Actinobacteria;o__Bifidobacteriales;f__Bifidobacteriaceae;g__Bifidobacterium;	0.017	0.004
k__Bacteria;p__Firmicutes;	0.007	0.003
k__Bacteria;p__Actinobacteria;c__Actinobacteria;o__Bifidobacteriales;f__Bifidob	0.006	0.011

Taxonomy	MiSeq A (%)	MiSeq B (%)
acteriaceae;g__Bifidobacterium;s__pseudolongum;		
k__Bacteria;p__Bacteroidetes;c__Bacteroidia;o__Bacteroidales;f__Bacteroidaceae;g__Bacteroides;	0.003	0.007
k__Bacteria;p__Firmicutes;c__Clostridia;o__Clostridiales;f__Eubacteriaceae;g__Anaerofustis;	0.003	0.000
k__Bacteria;p__Firmicutes;c__Clostridia;o__Clostridiales;f__Lachnospiraceae;g__Blautia;	0.003	0.000
k__Bacteria;p__Proteobacteria;c__Betaproteobacteria;	0.003	0.003
k__Bacteria;p__Bacteroidetes;c__Bacteroidia;o__Bacteroidales;f__Bacteroidaceae;g__Bacteroides;s__ovatus;	0.002	0.000
k__Bacteria;p__Cyanobacteria;c__Chloroplast;o__Streptophyta;	0.002	0.007
k__Bacteria;p__Firmicutes;c__Clostridia;o__Clostridiales;f__Christensenellaceae;g__Christensenella;	0.002	0.003
k__Bacteria;p__Proteobacteria;c__Alphaproteobacteria;o__Rickettsiales;f__mitochondria;	0.002	0.025
k__Bacteria;p__Proteobacteria;c__Gammaproteobacteria;o__Enterobacteriales;f__Enterobacteriaceae;	0.002	0.009
k__Bacteria;p__Actinobacteria;c__Actinobacteria;o__Actinomycetales;f__Pseudonocardiaceae;g__Actinomycetospora;	0.000	0.002
k__Bacteria;p__Actinobacteria;c__Actinobacteria;o__Bifidobacteriales;f__Bifidobacteriaceae;	0.000	0.002
k__Bacteria;p__Firmicutes;c__Bacilli;o__Lactobacillales;f__Streptococcaceae;g__Streptococcus;	0.000	0.002
k__Bacteria;p__Proteobacteria;c__Alphaproteobacteria;o__Rhizobiales;f__Methylobacteriaceae;	0.000	0.002
k__Bacteria;p__Proteobacteria;c__Alphaproteobacteria;o__Sphingomonadales;f__Sphingomonadaceae;	0.000	0.002

Taxonomy	MiSeq A (%)	MiSeq B (%)
k__Bacteria;p__Proteobacteria;c__Alphaproteobacteria;o__Sphingomonadales;f__Sphingomonadaceae;g__Sphingomonas;	0.000	0.010
k__Bacteria;p__Proteobacteria;c__Gammaproteobacteria;o__Pseudomonadales;f__Moraxellaceae;g__Acinetobacter;	0.000	0.004
k__Bacteria;p__Proteobacteria;c__Gammaproteobacteria;o__Pseudomonadales;f__Moraxellaceae;g__Acinetobacter;s__rhizosphaerae	0.000	0.002
k__Bacteria;p__Proteobacteria;c__Gammaproteobacteria;o__Xanthomonadales;f__Xanthomonadaceae;	0.000	0.002

Supplemental Table S2. Relative abundance of phylotypes detected by MinION^{TM} sequencer.

Taxonomy	MinION A (%)	MinION B (%)
k__Bacteria;p__Bacteroidetes;c__Bacteroidia;o__Bacteroidales;f__S24-7;	57.640	56.168
k__Bacteria;p__Firmicutes;c__Clostridia;o__Clostridiales;	10.641	11.355
k__Bacteria;p__Firmicutes;c__Erysipelotrichi;o__Erysipelotrichales;f__Erysipelotrichaceae;g__Allobaculum;	7.750	8.692
k__Bacteria;p__Firmicutes;c__Bacilli;o__Lactobacillales;f__Lactobacillaceae;g__Lactobacillus;	3.166	2.944
k__Bacteria;p__Bacteroidetes;c__Bacteroidia;o__Bacteroidales;f__Bacteroidaceae;g__Bacteroides;s__acidifaciens;	2.172	2.383
k__Bacteria;p__Proteobacteria;c__Betaproteobacteria;o__Burkholderiales;f__Alcaligenaceae;g__Sutterella;	1.970	2.103
k__Bacteria;p__Bacteroidetes;c__Bacteroidia;o__Bacteroidales;f__Rikenellaceae;	1.491	1.636
k__Bacteria;p__Firmicutes;c__Clostridia;o__Clostridiales;f__Lachnospiraceae;	1.473	1.822
k__Bacteria;p__Tenericutes;c__Mollicutes;o__RF39;	1.178	1.028
k__Bacteria;p__Cyanobacteria;c__4C0d-2;o__YS2;	1.141	1.308
k__Bacteria;p__Firmicutes;c__Clostridia;o__Clostridiales;f__Lachnospiraceae;g__[Ruminococcus];s__gnavus;	1.049	0.841
k__Bacteria;p__Firmicutes;c__Clostridia;o__Clostridiales;f__Ruminococcaceae;g__Oscillospira;	0.773	0.607
k__Bacteria;p__Firmicutes;c__Clostridia;o__Clostridiales;f__Ruminococcaceae;g__Ruminococcus;s__flavefaciens;	0.736	0.654
k__Bacteria;p__Firmicutes;c__Bacilli;o__Turicibacterales;f__Turicibacteraceae;g__Turicibacter;	0.681	0.561
k__Bacteria;p__Bacteroidetes;c__Bacteroidia;o__Bacteroidales;f__Bacteroidaceae;g__Bacteroides;	0.681	0.374

Taxonomy	MinION A (%)	MinION B (%)
k__Bacteria;p__Firmicutes;c__Clostridia;o__Clostridiales;f__Lachnospiraceae;g__Dorea;	0.663	0.794
k__Bacteria;p__Proteobacteria;c__Alphaproteobacteria;	0.663	0.607
k__Bacteria;p__Bacteroidetes;c__Bacteroidia;o__Bacteroidales;f__Porphyromonadaceae;g__Parabacteroides;	0.626	0.561
k__Bacteria;p__Firmicutes;c__Clostridia;o__Clostridiales;f__Ruminococcaceae;	0.571	0.841
k__Bacteria;p__Actinobacteria;c__Actinobacteria;o__Bifidobacteriales;f__Bifidobacteriaceae;g__Bifidobacterium;	0.552	0.701
k__Bacteria;p__Actinobacteria;c__Coriobacteriia;o__Coriobacteriales;f__Coriobacteriaceae;	0.534	0.701
k__Bacteria;p__Proteobacteria;c__Alphaproteobacteria;o__RF32;	0.479	0.607
k__Bacteria;p__Firmicutes;c__Erysipelotrichi;o__Erysipelotrichales;f__Erysipelotrichaceae;	0.460	0.280
k__Bacteria;p__Firmicutes;c__Clostridia;o__Clostridiales;f__Lachnospiraceae;g__Coprococcus;	0.368	0.327
k__Bacteria;p__Actinobacteria;c__Actinobacteria;o__Bifidobacteriales;f__Bifidobacteriaceae;g__Bifidobacterium;s__pseudolongum;	0.350	0.280
k__Bacteria;p__Firmicutes;c__Clostridia;o__Clostridiales;f__Ruminococcaceae;g__Ruminococcus;	0.313	0.421
k__Bacteria;p__Firmicutes;c__Clostridia;o__Clostridiales;f__Lachnospiraceae;g__Anaerostipes;	0.221	0.234
k__Bacteria;p__TM7;c__TM7-3;o__CW040;f__F16;	0.203	0.280
k__Bacteria;p__Bacteroidetes;c__Bacteroidia;o__Bacteroidales;	0.184	0.000
k__Bacteria;p__Tenericutes;c__Mollicutes;o__Anaeroplasmatales;f__Anaeroplasmataceae;g__Anaeroplasma;	0.147	0.000
k__Bacteria;p__Firmicutes;c__Clostridia;o__Clostridiales;f__Dehalobacteriaceae;g__Dehalobacterium;	0.147	0.093

Taxonomy	MinION A (%)	MinION B (%)
k__Bacteria;p__Firmicutes;c__Clostridia;o__Clostridiales;f__Peptococcaceae;g__rc4-4;	0.129	0.140
k__Bacteria;p__Firmicutes;c__Erysipelotrichi;o__Erysipelotrichales;f__Erysipelotrichaceae;g__Coprobacillus;	0.110	0.187
k__Bacteria;p__Firmicutes;c__Clostridia;o__Clostridiales;f__Lachnospiraceae;g__Blautia;	0.092	0.093
k__Bacteria;p__Firmicutes;c__Bacilli;o__Bacillales;f__Bacillaceae;g__Bacillus;	0.074	0.000
k__Bacteria;p__Bacteroidetes;c__Bacteroidia;o__Bacteroidales;f__Prevotellaceae;g__Prevotella;s__copri;	0.055	0.000
k__Bacteria;p__Firmicutes;c__Clostridia;o__Clostridiales;f__Lachnospiraceae;g__[Ruminococcus];	0.055	0.000
k__Bacteria;p__Actinobacteria;c__Actinobacteria;o__Bifidobacteriales;f__Bifidobacteriaceae;g__Bifidobacterium;s__animalis;	0.055	0.000
k__Bacteria;p__Bacteroidetes;c__Bacteroidia;o__Bacteroidales;f__Porphyromonadaceae;g__Parabacteroides;s__distasonis;	0.055	0.000
k__Bacteria;p__Proteobacteria;c__Deltaproteobacteria;o__Desulfovibrionales;f__Desulfovibrionaceae;g__Desulfovibrio;s__C21_c20;	0.055	0.000
k__Bacteria;p__Firmicutes;c__Clostridia;o__Clostridiales;f__Ruminococcaceae;g__Faecalibacterium;s__prausnitzii;	0.037	0.000
k__Bacteria;p__Bacteroidetes;c__Bacteroidia;o__Bacteroidales;f__Prevotellaceae;g__Prevotella;	0.037	0.000
k__Bacteria;p__Firmicutes;c__Erysipelotrichi;o__Erysipelotrichales;f__Erysipelotrichaceae;g__[Eubacterium];s__dolichum;	0.037	0.000
k__Bacteria;p__Firmicutes;c__Bacilli;o__Lactobacillales;f__Lactobacillaceae;g__Lactobacillus;s__reuteri;	0.037	0.000
k__Bacteria;p__Firmicutes;c__Clostridia;o__Clostridiales;f__Clostridiaceae;g__Clostridium;	0.037	0.187

Taxonomy	MinION A (%)	MinION B (%)
k__Bacteria;p__Bacteroidetes;c__Bacteroidia;o__Bacteroidales;f__Bacteroidaceae;g__Bacteroides;s__ovatus;	0.037	0.000
k__Bacteria;p__Verrucomicrobia;c__Verrucomicrobiae;o__Verrucomicrobiales;f__Verrucomicrobiaceae;g__Akkermansia;s__muciniphila;	0.037	0.093
k__Bacteria;p__Firmicutes;c__Erysipelotrichi;o__Erysipelotrichales;f__Erysipelotrichaceae;g__[Eubacterium];s__biforme;	0.037	0.000
k__Bacteria;p__Firmicutes;c__Clostridia;o__Clostridiales;f__[Mogibacteriaceae];	0.000	0.093
